# Neotropical ant-plant *Triplaris americana* attracts *Pseudomyrmex mordax* ant queens during seedling stages

**DOI:** 10.1007/s00040-017-0542-2

**Published:** 2017-02-06

**Authors:** María Fernanda Torres, Adriana Sanchez

**Affiliations:** 1 Ashworth Laboratories, Institute of Evolutionary Biology, University of Edinburgh, Edinburgh, UK; 2 Programa de Biología, Universidad del Rosario, Carrera 24 No. 63C-69, Bogotá, Colombia

**Keywords:** Ant–plant associations, Colombia, Colony founding, Host recognition, *Pseudomyrmex mordax*, *Triplaris americana*

## Abstract

The association between the myrmecophyte *Triplaris* and ants of the genus *Pseudomyrmex* is an often-reported example of mutualism in the Neotropics. The ants colonize the hollow stems of their hosts, and in exchange, the plants benefit from a reduced degree of herbivory. The previous studies have shown that workers can discriminate their host from other plants, including a closely related species. Little is known about how queens locate their host during the colonization process, but it has been suggested that host recognition is mediated by volatiles. Since queens of *Pseudomyrmex mordax* colonize their hosts during the seedling stage, we hypothesized that queens would discriminate leaves of seedlings from adult plants. To evaluate our hypothesis, we used a two-sided olfactometer, to test the preference of queens towards different leaf and plant ages of *Triplaris americana*. Virgin queens of *Pseudomyrmex mordax* preferred seedlings over adult plants, as well as plant leaves over empty controls, showing no discrimination for leaf age. Our results suggest that the volatiles virgin queens recognize are either produced or are more abundant at the early growing stage of the host when colonization is crucial for the host's survival.

## Introduction

In myrmecophytic associations, ant-plants provide nesting space inside domatia, and in some cases, food rewards, while ants protect plants against herbivory, pathogens, and encroaching vegetation (Rico-Gray and Oliveira 2007; Mayer et al. 2014; Sanchez and Bellota 2015). Myrmecophytism is widespread and very diverse in the Tropics where at least 100 different plant genera are inhabited by ant colonies (Davidson and McKey 1993; Chomicki and Renner 2015). One of the most conspicuous ant–plant mutualisms in the Neotropics is the association between *Pseudomyrmex* and *Triplaris*, known by the aggressive and stinging behavior of *Pseudomyrmex* (Oliveira et al. 1987; Davidson et al. 1988; Larrea-Alcázar and Simonetti 2007; Weir et al. 2012; Sanchez and Bellota 2015).

In order for myrmecophytic associations to be successful, recognition and host location mechanisms are required for the horizontal transmission of both partners (Clement et al. 2008; Blatrix and Mayer 2010). In cases, such as the mutualism between *Triplaris-Pseudomyrmex*, ant queens leave their colony in a nuptial flight to find a new host seedling and, subsequently, establish a colony inside the plant (Sanchez 2016). Establishment of the mutualism in the early stages of plant ontogeny reduces plants’ vulnerability to herbivory (Jürgens et al. 2006; Trager and Bruna 2006). Likewise, the longer it takes for the queen to find a host, the higher the rates of mortality during mating, dispersal, and colony foundation (Longino 1989; Frederickson 2006; Sanchez 2016). Consequently, effective host recognition is crucial in the establishment of the association and it has been shown that this recognition is primarily based on olfactory cues (Inui et al. 2001; Jürgens et al. 2006; Edwards et al. 2006; Dáttilo et al. 2009; Grangier et al. 2009).

Many authors have suggested that the communication in ant–plant interactions is promoted by plant volatile emissions, which contain information about host direction and distance (Djiéto-Lordon and Dejean 1999; Inui et al. 2001; Heil and McKey 2003; Edwards et al. 2006). Olfaction choice experiments testing species-specificity between *Cordia nodosa* and *Allomerus* and *Azteca* ants have demonstrated ants’ attraction toward volatiles released by hosts rather than those released by other plants (Edwards et al. 2006). Volatiles also function as indirect defense by attracting ants to a damaged area of the plant and more often towards younger and more vulnerable parts (Agrawal and Dubin-Thaler 1999; Brouat et al. 2000; Edwards et al. 2007; Mayer et al. 2008; Schatz et al. 2009; Blatrix and Mayer 2010). A recent study by Weir et al. (2012) showed that workers of *P. triplarinus* pruned non-host plants, discriminating between their host (*T. americana*) and other unrelated plants species and even selectively removed leaves of a closely related species, *T. poeppigiana* (which only hosts *Azteca*; Sanchez 2015). Their study also showed that ants and leaves of *T. americana* share some cuticular hydrocarbon and non-hydrocarbon components, which could be important for host discrimination. Volatile compounds may, therefore, allow the rapid and effective response of *Pseudomyrmex* against herbivores (Sanchez and Bellota 2015) and host discrimination related to pruning behavior (Davidson et al. 1988; Weir et al. 2012).

Production of volatile compounds may vary across different ontogenetic phases of the plant (Brouat et al. 2000; Edwards et al. 2007; Barton and Koricheva 2010; de Queiroz et al. 2013; Latteman et al. 2014). It is possible that queens can perceive those differences helping them distinguish between seedlings and adult plants; this, in turn, can increase the chances of an early successful colonization. The previous work has shown that *Pseudomyrmex* workers discriminate their *Triplaris* host from other species (Weir et al. 2012), but how *Pseudomyrmex* queens choose and locate their host is yet to be studied. It is not uncommon to find *Pseudomyrmex* queens inhabiting *Triplaris* at the seedling stage (Schremmer 1984; Sanchez 2016), which suggests that colonization occurs early. In our experiment, we tested the hypothesis that queens discriminate leaves of seedlings from adult plants. To do so, we used olfactory assays to investigate: (1) the possible attraction of *P. mordax* queens towards volatiles produced by leaves of *T. americana* and (2) whether queens could discriminate between leaf and/or plant age.

## Materials and methods

### Study site and species investigated

Plant and ant material were collected close to Quebrada Hemayacito, near to Pringamosal-Guamo, Tolima, Colombia (04°02′49.2′′N, 74°59′36.9′′W at 326 m). Historical climatic data from 1981 to 2010, obtained from the metereological station of the “IDEAM” (Instituto de Hidrología, Meteorología y Estudios Ambientales de Colombia) at Guamo, indicate that the annual mean temperature for the area is 27.9 °C. The warmest month is August (29.3 °C) and the coolest, November (27.1 °C). The annual mean maximum temperature is 33 °C and the minimum 22.7 °C. Annual mean precipitation is 1420 mm and relative humidity 74%.

**Fig. 1:**
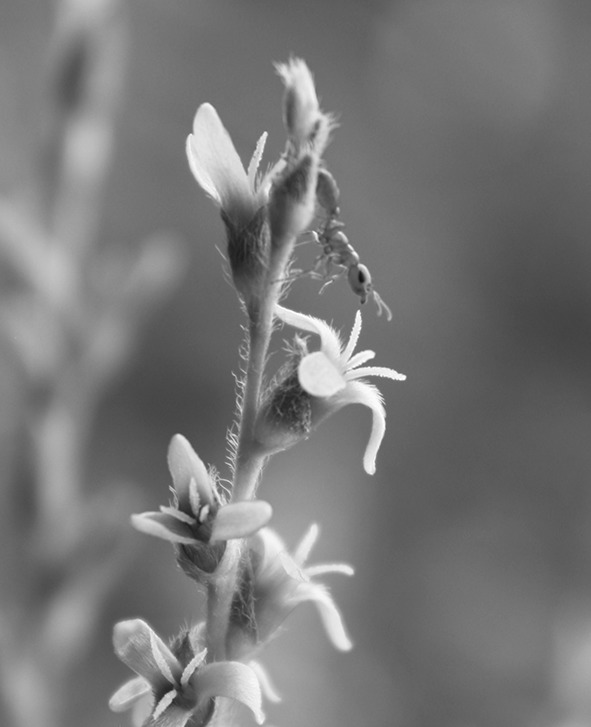
*Pseudomyrmex mordax* worker ant patrolling over a *Triplaris americana* inflorescence

*Triplaris* Loefl. (Polygonaceae) is a myrmecophytic genus of pioneer plants comprised of 18 species of trees, distributed from Mexico to Bolivia and Brazil (Brandbyge 1986, 1990). A conspicuous feature of the genus is that each plant individual harbors one ant colony in their hollow stems (Schremmer 1984; Sanchez 2015). *Triplaris americana* L. is the most common and widespread species in its genus, ranging from Panama to Bolivia and Brazil (Brandbyge 1986). *Pseudomyrmex* (Formicidae, Pseudomyrmicinae) comprises ca. 200 species distributed in the New World (Ward and Downie 2005), several of which form associations with distantly related host myrmecophytes (e.g., *Cordia, Tachigali, Triplaris, Vachellia*; Ward 1991, 1999). However, the *Pseudomyrmex triplarinus* subgroup is considered to nest exclusively in *Triplaris* (Ward 1999). *Pseudomyrmex mordax* (Warming 1894) is an obligate mutualist of *Triplaris* and it is known to be associated with only three host species (*T. americana, T. cumingiana and T. purdiei*) on a limited geographical distribution in Panama, Colombia, and Venezuela (Ward 1999; Sanchez 2015). At the study site, *T. americana* is the only known host available for *P. mordax* (Fig. 1).

Ant–plants in the genus *Triplaris* (Polygonaceae) are generally associated with *Pseudomyrmex* (Ward 1999) and, more rarely, with *Camponotus, Crematogaster* and *Azteca* (Oliveira et al. 1987; Sanchez 2015). Unlike many plant species that provide their associates with food structures (Davidson and McKey 1993; Chomicki and Renner 2015), in the case of the *Triplaris*-*Pseudomyrmex* association, food rewards are obtained via a third partner or sap-feeding trophobiont. The trophobionts are scale insects and/or mealybugs, principally members of the order Hemiptera (Sternorrhyncha: Coccoidea), which provide food rewards in the form of honeydew (Schremmer 1984; Oliveira et al. 1987; Davidson and McKey 1993; Ward 1999; Gonzalez-Teuber and Heil 2009; Sanchez 2016). It has also been suggested that parenchymal tissue and fungi may be alternative food sources for the colonies (Schremmer 1984; Defossez et al. 2009; Valverde and Hanson 2011; Blatrix et al. 2012).

The ant colony effectively defends the plant host by patrolling and attacking insect herbivores, reducing herbivory by as much as 16% (Sanchez and Bellota 2015). For the plant, the effectiveness of the ant defense depends on how early the colony establishes; for the ants, the earlier it can detect the plant signal, the higher the chances of successful colonization (Jürgens et al. 2006; Trager and Bruna 2006). However, with the exception of a recent host recognition study using *P. triplarinus* workers (Weir et al. 2012), preferences of young queens in the *Pseudomyrmex*–*Triplaris* association have not been studied.

### Queen discrimination assays

In this study we carried out olfactory assays to understand if young queens had a differential response to volatiles depending on leaf and/or plant age. Assays took place from June 2009 to March 2010. For the experiments, leaves were gently cleaned with a dry soft tissue to remove dust and then classified into four groups according to plant age (seedling vs. adult, randomizing leaf age) or to leaf age (young vs. mature leaf, randomizing plant age). Leaves were collected from plants within an approximate area of 1 km^2^, and to classify the leaves, individuals of *T. americana* were categorized as seedlings (<50 cm height and unbranched) or adult plants (>2 m). Young leaves, light green or reddish in color, corresponded to the first leaf or second leaf in any given branch; mature, dark green leaves were the most proximal in a branch. The material we used for the experiments showed no signs of herbivory or visible damage. Virgin alate queens of *P. mordax* were collected from the youngest branches of *T. americana* and then placed in vials for 1 day before the experiments. To avoid bias, queens were collected from different plants than the ones used for the assays. Experiments were conducted during a time span of 80 days.

**Fig. 2:**
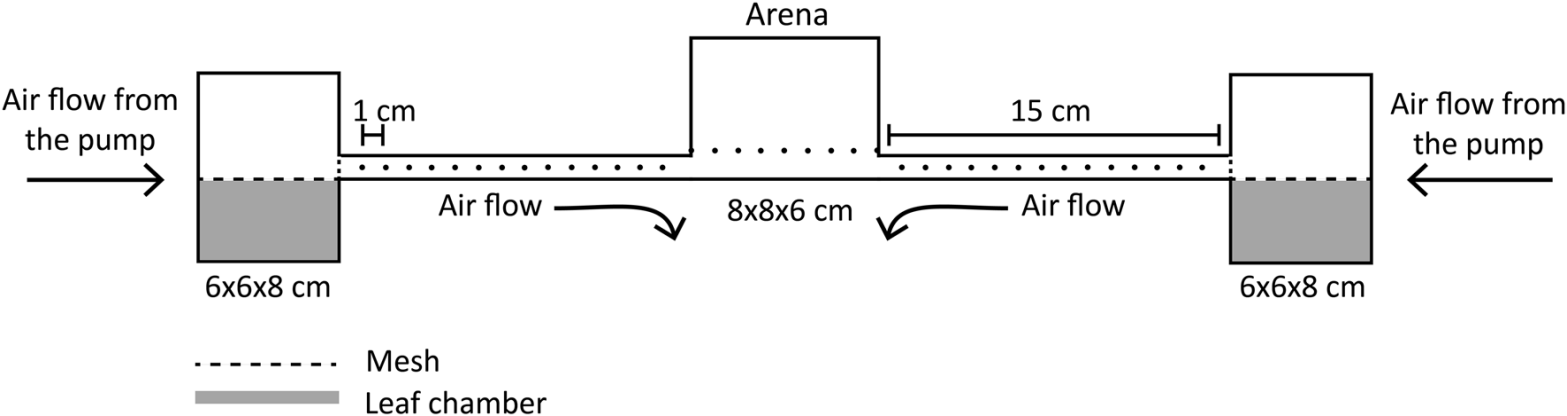
Diagram of the olfactometer used to test *Pseudomyrmex mordax* queen’s preference

An olfactometer, consisting of a central arena (hereafter referred to as an arena), connected to two lateral boxes (each of 6 × 8 × 6 cm) was used to carry out the scent attraction experiments (García-Robledo and Horvitz 2009). Polypropylene tubes (15 cm long) connected the lateral boxes to the arena (8 × 6 × 8 cm) and small holes (1 mm in diameter at 1 cm intervals) were made along these tubes to allow airflow and prevent volatile mixing. An air pump, connected to the lateral boxes, injected air into the arena at a rate of 100 ml min^−1^ (Fig. 2). Parts were sealed together with Parafilm to prevent interference from glue scent. Meshes were placed at each end of the tube to avoid visual and physical contact between the queens and the plant material.

Ant queens were deposited in the arena and the same number of leaves was placed in the lateral boxes according to the treatment. Queens were presented with one of two treatments: (1) plant age, consisting of leaves from seedlings and adult plants, plus controls (empty box). (2) Leaf age, consisting of young and mature leaves, plus controls. To prevent exhaustion and stress, each queen was subjected to only one treatment assay (plant age or leaf age, but not both) with its respective three trials (i.e., seedling vs. adult, seedling vs. control, and adult vs. control) and only one trial per day. For each queen trial, we randomized assay order and the box that would contain a certain type of leaf or control. After each trial, we cleaned the olfactometer with 96% ethanol and left to dry for 10 min (Hulcr et al. 2011). New plant material was used for each trial.

Every trial lasted 10 min; the first 2 min were used for queen acclimation and exploration, while the remaining 8 min were recorded as time (measured in seconds) spent by the queen on either side of the olfactometer (treatment preference). A preference coefficient (PC) was then estimated (Quiroz et al. 1997) as the ratio of the average preference in seconds between young/mature leaves, seedling/adult leaves, and leaves/control. Ratios greater or less than one indicated a preference, while a ratio equal or close to one indicated no difference in preference between the sides. Since the previous studies have demonstrated that *Pseudomyrmex* colonies can distinguish their host from closely related species (*T. poeppigiana*) and other plants growing nearby (Davidson et al. 1988; Weir et al. 2012), this experimental design focused on the ability of the queens to discriminate between their host’s plant and leaf ages.

A total of 101 and 100 ant queens were used for plant and leaf age assays, respectively. Because our data were continuous (we measured time spent by queens in s) instead of discrete (just recording queen's choice), not all data sets were normal. We tested the difference in queens’ preference on each treatment using the non-parametric Friedman test in the stats R package version 3.2.0 (R Core Team 2015) combined with a *post hoc* pairwise comparison using the Nemenyi multiple comparison test implemented in the PMCMR package (Pohlert 2014). To visualize the amount of time queens spent on each side of the olfactometer, a violin plot was done using the seaborn v0.7.1 Python library (Waskom et al. 2016).

## Results

**Fig. 3:**
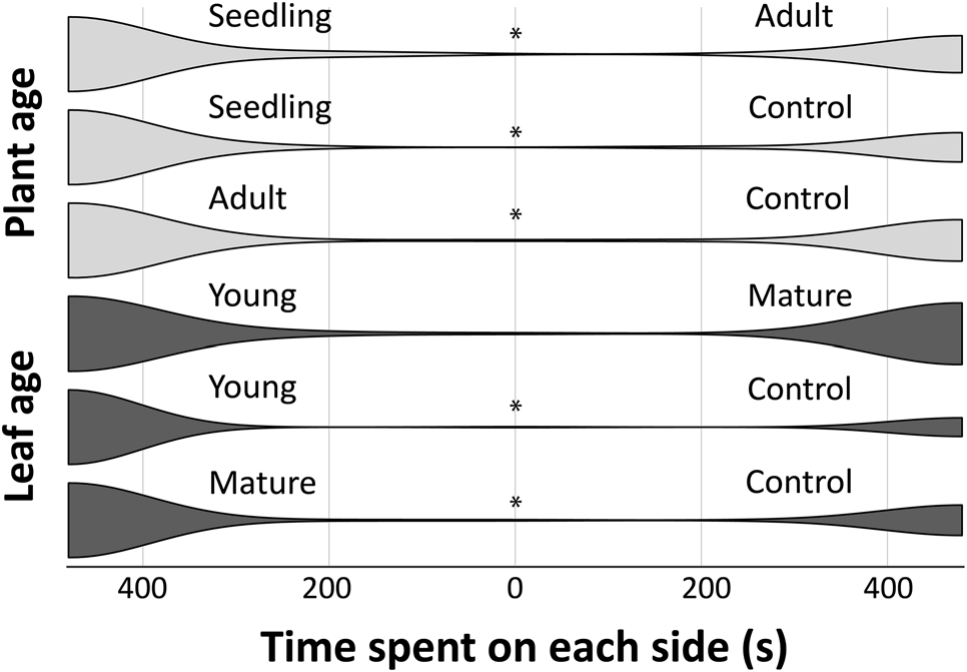
Violin plot of the distribution of time (s) spent by *Pseudomyrmex mordax* queens on each side of the olfactometer. *Asterisks* indicate significant differences on queens’ preference

Results from the Friedman test report significant differences within plant (*χ* ^2^ = 61.75, *df* = 5, *P* < 0.001) and leaf (*χ* ^2^ = 91.86, *df* = 5, *P* < 0.001) age treatments (Table 1). Within treatments, *Pseudomyrmex mordax* queens spent significantly more time at the boxes containing *T. americana* leaves, regardless of plant or leaf age, when compared to an empty box (control; Fig. 3). For the plant age treatment assays, queens spent, on average, more time on the side containing seedling and adult leaves (342 s, PC = 2.46 and 340 s, PC = 1.77, respectively) than on the controls (Fig. 3; Table 1). Queens also showed a significant preference for seedlings compared to adult plants (312± vs 160± s, respectively; PC = 1.96).

**Table 1:** Results of the Friedman non-parametric test and post hoc Nemenyi multiple comparisons for the preferences of *Pseudomyrmex mordax* queens

Treatment	Assay	*P* value
Plant age	Seedling vs. adult	0.003*
	Seedling vs. empty	>0.0001**
	Adult vs. empty	0.009*
Leaf age	Young vs. mature	0.085
	Young vs. empty	>0.0001**
	Mature vs. empty	>0.0001**

*P < 0.05, **P < 0.001

For the leaf age treatment assays, no significant differences were detected between the time spent on young and mature leaves from random trees (265 and 215 s, respectively; PC = 1.23), but queens spent significantly more time on young (384 s, PC = 3.98) and mature leaves (339 s PC = 2.4) when compared to the control (Fig. 3; Table 1). Thus, queens showed no significant discrimination between young or mature leaves, but they had a significant preference for seedlings over adult plants.

## Discussion

We tested the capacity of *P. mordax* queens to distinguish volatiles from different leaf and plant ages. Our results show that virgin queens of *P. mordax* are attracted to volatiles produced by *T. americana* leaves, especially to the ones produced during the seedling stage (Fig. 3). Preference of *P. mordax* queens for seedling leaves instead of those from adult plants is an indicator of ontogenetic differences in the leaves’ chemical composition (either in relative abundances or presence/absence) that could be shaping the cues that allow host recognition. Preliminary results indicate that differences between seedlings and adults may be attributable to changes in the relative abundance of volatiles (M. F. Torres unpubl. data) as has been found in other studies (Brouat et al. 2000). Furthermore, the presence and concentration of these compounds can vary over time according to their function, as it has been shown to occur in the case of attraction (Proffit et al. 2008).

Other ant-plants are known to use volatiles as cues to recruit workers in response to herbivore attacks as well as to attract foundress queens (Inui et al. 2001; Bruce et al. 2005; Jürgens et al. 2006; Blatrix and Mayer 2010 and references therein; Orona-Tamayo and Heil 2013). As shown by Weir et al. (2012), worker ants of *P. triplarinus* discriminate between their host and other common plant species, including a closely related species (*T. poeppigiana*). Although the main focus of the study was on pruning behavior and not colonization, the authors propose that the primary chemical signals involved in host discrimination are hydrocarbon and non-hydrocarbon cuticular leaf compounds which, in many instances, are shared between the ants and *T. americana* leaves. Other myrmecophytic ants have similar abilities to recognize and discriminate among compounds widely present in different plant species and in different proportions (Seidel et al. 1990; Brouat et al. 2000; Bruce et al. 2005; Blatrix and Mayer 2010). Studies in other insects, such as *Leptinotarsa decemlineata*, demonstrate that subtle changes in the proportions of (3*E*)-hexenol, (2*E*)-hexenol, (2*Z*)-hexenol, and (2*E*)-hexenal alter their response from attraction to repellency towards the host (Visser and Ave 1978). In addition, both common and uncommon compounds play an important role in recognition. For example, the release of six-carbon molecules and common defense volatiles could provide general information about host location, reducing the search range to a subset of plants in a smaller area (Bruce et al. 2005). Therefore, one or few more specific compounds may indicate the queen the precise location of the host to queens. Such specificity towards host-plant cues means that the presence of non-host plants and local environmental conditions would not influence the host’s bouquet (Harrewijn et al. 1995). Nevertheless, volatiles are not the only cues: visual, touch, and taste signs are also involved in the final process of recognition, when foundress queens are in direct contact with the plant (Visser 1988; Weir et al. 2012). In addition, as demonstrated by Dáttilo et al. (2009), some non-volatile compounds produced by non-host plants act as repellents to ant queens.

The ability to distinguish between plant development stages, along with the use of chemical cues to find a mutualist plant partner, suggests that the early establishment of the mutualism has been favored through the evolution of myrmecophytism (Nishi and Romero 2008; Sanchez 2016). In ant–plant mutualisms, nesting space is a limiting factor for ant colony size (Fonseca 1993) and colony size correlates with defense effectiveness (Heil and McKey 2003; Brouat and McKey 2001; Frederickson et al. 2012). However, even when myrmecophytes are at the seedling stage, it has been shown that the first hatched workers of the incipient colony patrol the plant (Brouat and McKey 2000; Moog et al. 2005). It has also been shown that some ant-plants do not produce chemical defenses, but instead invest energy and resources in the production of domatia to house more ants (Trager and Bruna 2006). As a pioneer plant, *T. americana* invests much of its energy in growth; as a consequence, there is a rapid increase in the amount of available space for the growing ant colony, and therefore, there is more protection to the plant host (Oliveira et al. 1987; Sanchez 2016). Hence, despite of seedlings providing little nesting space and the size of the ant colony being small, the early establishment ensures queen’s survival and protection to the host, and reduces the chances of host colonization by a parasitic ant species (Frederickson 2006; Sanchez 2016). This can be achieved with the early production of attractive signals.

## Conclusion

Although a previous study has shown that workers in the ant-plant interaction between *Triplaris* and *Pseudomyrmex* recognize and discriminate their host from other plant species (Weir et al. 2012), we are not aware of any studies that have explored if queens discriminate between adult and seedling plants. *Pseudormyrmex mordax* queens were attracted to volatiles of *T. americana* leaves, especially those of seedlings. Differences in the relative abundance of volatiles may provide a cue for founding queens and further studies involving a detailed chemical analysis of the compounds constituting the bouquet of *T. americana* are recommended.

## References

[CR1] Agrawal AA, Dubin-Thaler BJ (1999). Induced responses to herbivory in the Neotropical ant-plant association between *Azteca* ants and *Cecropia* trees: response of ants to potential inducing cues. Behav Ecol Sociobiol.

[CR2] Barton KE, Koricheva J (2010). The ontogeny of plant defense and herbivory: characterizing general patterns using meta-analysis. Am Nat.

[CR3] Blatrix R, Mayer V, Baluška F, Ninkovic V (2010). Communication in ant-plant symbioses. Plant communication from an ecological perspective.

[CR4] Blatrix R, Djiéto-Lordon C, Mondolot L, La Fisca P, Voglmayr H, McKey D (2012). Plant-ants use symbiotic fungi as a food source: new insight into the nutritional ecology of ant-plant interactions. Proc Roy Soc Lond B Bio.

[CR5] Brandbyge J (1986). A revision of the genus *Triplaris* (Polygonaceae). Nord J Bot.

[CR6] Brandbyge J (1990). Woody Polygonaceae from Brazil: new species and a new interpretation. Nord J Bot.

[CR7] Brouat C, McKey D (2000). Origin of caulinary ant domatia and timing of their onset in plant ontogeny: evolution of a key trait in horizontally transmitted ant-plant symbioses. Biol J Linn Soc.

[CR8] Brouat C, McKey D, Bessière JM, Pascal L, Hossaert-McKey M (2000). Leaf volatile compounds and the distribution of ant patrolling in an ant-plant protection mutualism: Preliminary results on *Leonardoxa* (Fabaceae: Caesalpinioideae) and *Petalomyrmex* (Formicidae: Formicinae). Acta Oecol.

[CR9] Brouat C, McKey D (2001). Leaf-stem allometry, hollow stems, and the evolution of caulinary domatia in myrmecophytes. New Phytol.

[CR10] Bruce TJA, Wadhams LJ, Woodcock CM (2005). Insect host location: a volatile situation. Trends Plant Sci.

[CR11] Clement LW, Köppen SCW, Brand WA, Heil M (2008). Strategies of a parasite of the ant-*Acacia* mutualism. Behav Ecol Sociobiol.

[CR12] Chomicki G, Renner SS (2015). Phylogenetics and molecular clocks reveal the repeated evolution of ant-plants after the late Miocene in Africa and the early Miocene in Australasia and the Neotropics. New Phytol.

[CR13] Dáttilo WFC, Izzo TJ, Inouye BD, Vasconcelos HL, Bruna EM (2009). Recognition of host plant volatiles by *Pheidole minutula* Mayr (Myrmecinae), an Amazonian Ant-Plant specialist. Biotropica.

[CR14] Davidson DW, Longino JT, Snelling RR (1988). Pruning of host plant neighbors by ants: an experimental approach. Ecology.

[CR15] Davidson DW, McKey D (1993). The evolutionary ecology of symbiotic ant-plant relationships. J Hymenop Res.

[CR16] Defossez E, Selosse MA, Dubois MP, Mondolot L, Faccio A, Djieto-Lordon C, McKey D, Blatrix R (2009). Ant-plants and fungi: a new threeway symbiosis. New Phytol.

[CR17] Djiéto-Lordon C, Dejean A (1999). Innate attraction supplants experience during host plant selection in an obligate plant-ant. Behav Processes.

[CR42] de Queiroz ACM, da Costa FV, de Siqueira-Neves F, Fagundes M (2013). Does leaf ontogeny lead to changes in defensive strategies against insect herbivores?. Arthropod-Plant Inte.

[CR18] Edwards DP, Hassall M, Sutherland WJ, Yu DW (2006). Assembling a mutualism: ant symbionts locate their host plants by detecting volatile chemicals. Insect Soc.

[CR19] Edwards DP, Arauco R, Hassall M, Sutherland WJ, Chamberlain K, Wadhams LJ, Douglas WY (2007). Protection in an ant-plant mutualism: an adaptation or a sensory trap?. Anim Behav.

[CR20] Frederickson ME (2006). The reproductive phenology of an Amazonian ant species reflects the seasonal availability of its nest sites. Oecologia.

[CR21] Frederickson ME, Ravenscraft A, Miller GA, Hernández LMA, Booth G, Pierce NE (2012). The direct and ecological costs of an ant-plant symbiosis. Am Nat.

[CR22] Fonseca CR (1993). Nesting space limits colony size of the plant-ant *Pseudomyrmex concolor*. Oikos.

[CR23] García-Robledo C, Horvitz CC (2009). Host plant scents attract rolled-leaf beetles to Neotropical gingers in a Central American tropical rain forest. Entomol Exp Appl.

[CR24] Gonzalez-Teuber M, Heil M (2009). The role of extrafloral nectar amino acids for the preferences of facultative and obligate ant mutualists. J Chem Ecol.

[CR25] Grangier J, Dejean A, Malé PJG, Solano PJ, Orivel J (2009). Mechanisms driving the specificity of a myrmecophyte-ant association. Biol J Linn Soc.

[CR26] Harrewijn P, Minks AK, Mollema C (1995). Evolution of plant volatile production in insect-plant relationships. Chemoecology.

[CR27] Heil M, McKey D (2003). Protective ant-plant interactions as model systems in ecological and evolutionary research. Annu Rev Ecol Evol S.

[CR28] Hulcr J, Mann R, Stelinski LL (2011). The scent of a partner: ambrosia beetles are attracted to volatiles from their fungal symbionts. J Chem Ecol.

[CR29] Inui Y, Itioka T, Murase K, Yamaoka R, Itino T (2001). Chemical recognition of partner plant species by foundress ant queens in *Macaranga*-*Crematogaster* myrmecophytism. J Chem Ecol.

[CR30] Jürgens A, Feldhaar H, Feldmeyer B, Fiala B (2006). Chemical composition of leaf volatiles in *Macaranga* species (Euphorbiaceae) and their potential role as olfactory cues in host-localization of foundress queens of specific ant partners. Biochem Syst Ecol.

[CR31] Larrea-Alcázar DM, Simonetti JA (2007). Why are there few seedlings beneath the myrmecophyte *Triplaris americana*?. Acta Oecol.

[CR32] Latteman TA, Mead JE, DuVall MA, Bunting CC, Bevington JM (2014). Differences in anti-herbivore defenses in non-myrmecophyte and myrmecophyte *Cecropia* Trees. Biotropica.

[CR33] Longino JT (1989). Geographic variation and community structure in an ant-plant mutualism: *Azteca* and *Cecropia* in Costa Rica. Biotropica.

[CR34] Mayer V, Schaber D, Hadacek F (2008). Volatiles of myrmecophytic Piper plants signal stem tissue damage to inhabiting *Pheidole* ant-partners. J Ecol.

[CR35] Mayer VE, Frederickson ME, McKey D, Blatrix R (2014). Current issues in the evolutionary ecology of ant–plant symbioses. New Phytol.

[CR36] Moog J, Saw LG, Hashim R, Maschwitz U (2005). The triple alliance: how a plant-ant, living in an ant-plant, acquires the third partner, a scale insect. Insect Soc.

[CR37] Nishi AH, Romero GQ (2008). Colonization pattern of *Cecropia* by *Azteca* ants: influence of plant ontogeny, environment and host plant choice by queens. Sociobiology.

[CR38] Oliveira PS, Oliveira Filho AT, Cintra R (1987). Ant foraging on ant-inhabited *Triplaris* (Polygonaceae) in Western Brazil a field experiment using live termite baits. J Trop Ecol.

[CR39] Orona-Tamayo D, Heil M (2013). Stabilizing mutualisms threatened by exploiters: new insights from ant–plant research. Biotropica.

[CR40] Pohlert T (2014) The pairwise multiple comparison of mean ranks package (PMCMR). R package. http://CRAN.R-project.org/package=PMCMR. Accessed 4 Nov 2016

[CR41] Proffit M, Schatz B, Bessière JM, Chen C, Soler C, Hossaert-McKey M (2008). Signalling receptivity: Comparison of the emission of volatile compounds by figs of *Ficus hispida* before, during and after the phase of receptivity to pollinators. Symbiosis.

[CR43] Quiroz A, Pettersson J, Pickett JA, Wadhams LJ, Niemeyer HM (1997). Semiochemicals mediating spacing behavior of bird cherry-oat aphid, *Rhopalosiphum padi* feeding on cereals. J Chem Ecol.

[CR44] R Core Team (2015) R: A language and environment for statistical computing. R Foundation for Statistical Computing, Vienna. http://www.R-project.org/

[CR45] Rico-Gray V, Oliveira PS (2007). The ecology and evolution of ant-plant interactions.

[CR46] Sanchez A (2015). Fidelity and promiscuity in an ant-plant mutualism: a case study of *Triplaris* and *Pseudomyrmex*. PLoS One.

[CR47] Sanchez A, Bellota E (2015). Protection against herbivory in the mutualism between *Pseudomyrmex dendroicus* (Formicidae) and *Triplaris americana* (Polygonaceae). J Hymenopt Res.

[CR48] Sanchez A (2016). Establishing an ant-plant mutualism: Foundress queen mortality and acquiring the third partner. Insect Soc.

[CR49] Schatz B, Djiéto-Lordon C, Dormant L, Bessière JM, McKey C, Blatrix R (2009). A simple non-specific chemical signal mediates defence behaviour in a specialized ant-plant mutualism. Curr Biol.

[CR50] Schremmer F (1984). Untersuchungen und Beobachtungen zur Ökoethologie der Pflanzenameise *Pseudomyrmex triplarinus*, welche die Ameisenbäume der Gattung *Triplaris* bewohnt. Zool Jahrb Abt Anat Ontog Tiere.

[CR51] Seidel JL, Epstein WW, Davidson DW (1990). Neotropical ant gardens, I. Chemical constituents. J Chem Ecol.

[CR52] Trager MD, Bruna EM (2006). Effects of plant age, experimental nutrient addition and ant occupancy on herbivory in a neotropical myrmecophyte. J Ecol.

[CR53] Valverde JP, Hanson P (2011). Parenchyma: a neglected plant tissue in the *Cecropia*/ant mutualism. Symbiosis.

[CR54] Visser JH (1988). Host-plant finding by insects: Orientation, sensory input and search patterns. J Insect Physiol.

[CR55] Visser JH, Avé DA (1978). General green leaf volatiles in the olfactory orientation of the Colorado beetle, *Lerptinotarsa decemlineata*. Entomol Exp Appl.

[CR56] Waskom M, Botvinnik O, Drewokane, Hobson P, David, Halchenko Y, Lukauskas S, Cole JB, Warmenhoven J, de Ruiter J, Hoyer S, Vanderplas J, Villalba S, Kunter G, Quintero E, Martin M, Miles A, Meyer K, Augspurger T, Yarkoni T, Bachant P, Williams M, Evans C, Fitzgerald C, Brian, Wehner D, Hitz G, Ziegler E, Qalieh A, Lee A (2016) seaborn v0.7.0. https://doi.org/10.5281/zenodo.54844. Accessed 19 Dec 2016

[CR57] Ward PS, Huxley CR, Cutler DF (1991). Phylogenetic analysis of Pseudomyrmecine ants associated with domatia-bearing plants. Ant-plant interactions.

[CR58] Ward PS (1999). Systematics, biogeography and host plant associations of the *Pseudomyrmex viduus* group (Hymenoptera: Formicidae), *Triplaris*- and *Tachigali*-inhabiting ants. Zool J Linn Soc.

[CR59] Ward PS, Downie DA (2005). The ant subfamily Pseudomyrmecinae (Hymenoptera: Formicidae): phylogeny and evolution of big-eyed arboreal ants. Syst Entomol.

[CR60] Warming E (1894). Om et par af Myrer beboede Traeer. Videnskabelige Meddelelser fra den Naturhistoriske Forening i Kjøbenhaun.

[CR61] Weir TL, Newbold S, Vivanco JM, van Haren M, Fritchman C, Dossey AT, Bartram S, Boland W, Cosio EG, Kofer W (2012). Plant-inhabiting ant utilizes chemical cues for host discrimination. Biotropica.

